# Applying mathematical models to predict resident physician performance and alertness on traditional and novel work schedules

**DOI:** 10.1186/s12909-016-0751-9

**Published:** 2016-09-13

**Authors:** Elizabeth B. Klerman, Scott A. Beckett, Christopher P. Landrigan

**Affiliations:** 1 Division of Sleep and Circadian Disorders, Departments of Neurology and Medicine, Brigham and Women’s Hospital, Boston, MA 02115 USA; 2 Division of Sleep Medicine, Department of Medicine, Harvard Medical School, Boston, MA 02115 USA; 3 Division of General Pediatrics, Department of Medicine, Boston Children’s Hospital, Boston, MA 02115 USA

**Keywords:** Resident, Intern, Physician-in-training, Modeling, Sleep deprivation, Circadian misalignment

## Abstract

**Background:**

In 2011 the U.S. Accreditation Council for Graduate Medical Education began limiting first year resident physicians (interns) to shifts of ≤16 consecutive hours. Controversy persists regarding the effectiveness of this policy for reducing errors and accidents while promoting education and patient care. Using a mathematical model of the effects of circadian rhythms and length of time awake on objective performance and subjective alertness, we quantitatively compared predictions for traditional intern schedules to those that limit work to ≤ 16 consecutive hours.

**Methods:**

We simulated two traditional schedules and three novel schedules using the mathematical model. The traditional schedules had extended duration work shifts (≥24 h) with overnight work shifts every second shift (including every third night, Q3) or every third shift (including every fourth night, Q4) night; the novel schedules had two different cross-cover (XC) night team schedules (XC-V1 and XC-V2) and a Rapid Cycle Rotation (RCR) schedule. Predicted objective performance and subjective alertness for each work shift were computed for each individual’s schedule within a team and then combined for the team as a whole. Our primary outcome was the amount of time within a work shift during which a team’s model-predicted objective performance and subjective alertness were lower than that expected after 16 or 24 h of continuous wake in an otherwise rested individual.

**Results:**

The model predicted fewer hours with poor performance and alertness, especially during night-time work hours, for all three novel schedules than for either the traditional Q3 or Q4 schedules.

**Conclusions:**

Three proposed schedules that eliminate extended shifts may improve performance and alertness compared with traditional Q3 or Q4 schedules. Predicted times of worse performance and alertness were at night, which is also a time when supervision of trainees is lower. Mathematical modeling provides a quantitative comparison approach with potential to aid residency programs in schedule analysis and redesign.

## Background

Medical errors have been a leading cause of death in the United States for over a decade [1, 2]. Physician sleep deprivation increases the risk of accidents and patient health through medical errors, as well as physician health through the risk of needle stick injuries and motor vehicle crashes [3–15]. In response both to emerging research documenting the hazards of resident physicians’ sleep deprivation and to public concerns with this risk, the U.S. Accreditation Council for Graduate Medical Education (ACGME) initiated limitations on the number of consecutive hours a physician trainee can work for all residency programs in 2003 [16]. These limits, however, continued to allow residents to work for up to 30 consecutive hours every other shift (Q3), work 88 h per week (averaged over 4 weeks, permitting much longer hours), and to work 24 days in a row. Following a subsequent year-long investigation and literature review that uncovered particular concerns with the duration of resident physicians’ traditional extended work shifts, the U.S. Institute of Medicine recommended that residents’ schedules be further redesigned, such that no resident works more than 16 consecutive hours without sleep [12]. The ACGME, however, implemented a 16 h consecutive work limit for first year interns only (PGY1) in July of 2011 [16], and continued to allow PGY2 and more senior residents to work for up to 28 consecutive hours, 88 h per week averaged over 4 weeks, and 24 consecutive days in a row. This recommendation therefore produced little change in overall schedule hours. Residency program directors, who are not trained in work schedule design or sleep and circadian biology, must design their interns’ work schedule to provide around-the-clock coverage, while ensuring that no individual exceeds the maximum allowable number of hours. Little guidance and few tools exist to aid them as they attempt to do this.

Both circadian misalignment and increased wake duration (i.e., sleep deprivation) are associated with decreases in objective performance and subjective alertness (e.g., [17]). Mathematical models have been used to predict their effects on accidents. Simulation predictions from two mathematical models of performance correlate with train accident rate and cost [18] and with driving accidents [19]. Our goal was to develop a tool to facilitate resident program directors’ efforts to design evidence-based schedules that would: (1) predict an increase over current schedules in the performance and alertness of residents and (2) adhere to the IOM recommendations for intern work hour limits. Both objective performance and subjective alertness were simulated because the time-course of their response to sleep deprivation and recovery is different and because while objective performance decrements may be correlated with errors and accidents [3, 6, 8], the decision to stop a task (e.g., driving) and seek help or a countermeasure (e.g., caffeine, nap) is based on subjective alertness assessments, even though these self-assessments are known to be unreliable (e.g., [20]). To accomplish our goal, we applied methods developed initially to design NASA-related mission schedules to quantify and compare the effects of different resident work schedules [21, 22] on both the predicted performance and alertness of each individual on each schedule, and on the overall performance of the group working a particular schedule (i.e., the net objective performance and subjective alertness of the individual group members).

Since performance decrements related to shift work are associated with a change in the distribution (which may be more dramatic than a change in the average value) of cognitive responses to more time spent at lower performance levels [23], we focused on the lowest percentiles of predicted performance rather than the mean or median level. Our previous simulation work demonstrated that the lowest quartile of predicted performance is sensitive to scheduling changes that cause circadian misalignment [21].

## Methods

### Simulations

Performance and alertness have a non-linear response to circadian phase and length of time awake. While both worsen with extended wake and at circadian “night”, both objective performance and subjective alertness can also partially rebound during circadian day, even after an individual has been awake all night. This non-linear interaction makes prediction of the effects of varying schedules difficult. We choose to use two linked mathematical models to quantitatively predict the effects of different schedules on performance and alertness: a model of the effect of light on the circadian pacemaker and a model of the effects of circadian phase and length of time awake on human objective performance (as measured by “Cognitive Throughput”) and on subjective alertness (as measured by “Subjective Alertness”) [24–26]. The model of the effect of light on the circadian pacemaker is used to estimate circadian phase (timing) and amplitude based on the timing and intensity of light exposure. These models have been validated with data in experimental and field-based settings [27, 28] and have been shown to yield good predictions to the range of sleep-wake schedules and light levels encountered by training physicians [29, 30]. Our approach included tailoring our simulation and analysis techniques toward using a combined schedule representing all individuals collectively sharing the rotation rather than for one schedule for one individual.

Three novel and two traditional schedules were simulated with the Circadian Performance Simulation Software version 1.2 (CPSS) [31] using methods described in the CPSS User’s manual [32]. The CPSS is a software application that implements the mathematical models of the effect of light on the circadian pacemaker and linked models of performance and alertness described above [24, 31, 33]; it predicts performance and alertness for every minute that an individual is awake during the schedule. Output values range from 0 (worst) to 100 (best).

### Schedules

The schedules simulated in this paper are derived from research on resident schedule design performed by the Harvard Work Hours, Health, and Safety Group (HWHHSG), as well as novel schedules proposed by residents from the Boston Combined Residency Program in Pediatrics (BCRP) in response to the 2011 ACGME limitations [3, 5–8, 10, 14, 15, 25, 34–38]. Only new schedules consistent with the IOM 2011guidelines [16] were simulated. In addition, two traditional schedules in which day shifts alternate with overnight shifts every three (“Q3”) or four (“Q4”) days were simulated. These traditional schedules do not conform to the current guidelines for PGY1 residents, but are still permitted for PGY2 and higher residents.

The five groups of schedules of approximately one month (28 days) duration were: Q3, Q4, Rapid Cycle Rotation (RCR) [3, 6], Cross-Cover Version 1 (XC-V1) and Cross Cover Version 2 (XC-V2). Each group schedule is composed of 3 – 7 individual schedules: for example, 3 individual schedules are combined for a Q3 group schedule. Details of the individual schedules and the average amount of sleep on those schedules are presented in Table 1. All schedules were simulated beginning with one week of 7-h sleep episodes from 10:30 PM - 5:30 AM before the one month of work. This was done to reduce any transients from the initial conditions of the program (as recommended in [32]). The sleep schedules were designed to match the average amount of sleep reported by individuals on these schedules in applied working situations [6, 10].

**Table 1:** Schedule design and rules

Schedule	Basic work schedule	Shortened shifts:	Days off	NAP opportunities	Average sleep duration
Q3	Day 1 “Day”: 6:30 AM - 5:30 PM.	If a Call shift ends on a Saturday, Sunday, or Monday. work will end at 10 AM	If a Call shift ends on a Friday, Saturday, or Sunday, the following day will be a day off (instead of a Day 1 shift)	Recommended 3:30 AM - 4:30 AM during a Call shift.	6.5 hr
	Days 2-3 “Call: 6:30 AM - 12:30 PM the following day.				
Q4	Day 1 “Day”: 6:30 AM - 5:30 PM	If a Call shift ends on a Saturday, or Sunday. work will end at 10 AM	If a Call shift ends on a Friday, the following Saturday and Sunday will be days off (instead of a Day 1 and Day 2 shift)	Recommended 3:30 AM - 4:30 AM during a Call shift.	7.0 hr
	Day 2 “Day”: 6:30 AM - 5:30 PM				
	Days 3-4 “Call”: 6:30 AM - 12:30 PM the following day				
			If a Call shift ends on a Saturday the following Sunday will be a day off. (instead of a Day 1 shift)		
RCR	Day 1 “Day”: 6:30 AM - 5:30 PM.	[None]	If a Call shift ends on a Friday, Saturday, or Sunday, the following day will be a day off (instead of a Day 1 shift)	Recommended 3:30 AM - 4:30 AM during a Call shift.	7.5 hr
	Day 2 “Extended”: 6:30 AM - 10:00 PM				
	Days 3-4 “Overnight”: 9:30 PM - 1 PM the following day.			Recommended 3 PM - 5 PM before a Call shift.	
XC: Intern A + B (combines with XC-V1 or XC-V2)	All Days “Day”: 6:30 AM - 5:30 AM	[None]	Intern A: the 2nd and 4th weekend of every month	[None]	7.2 hr
			Intern B: the 1st and 3rd weekends of every month.		
XC-V1 (combines with XC InternA + B)	For 3 of the 4 weeks:	[None]	If a Day shift is on a Friday, Saturday, Sunday, that day is off	[None]	7.0 hr
	Days 1-5 “Day”: 7 AM - 5:30 PM then weekend off				
	For 1 of the 4 weeks:				
	Days 1-4 or Days 1-5 “Overnight”: 5 PM - 7:30 AM the following day				
XC-V2 (combines with XC InternA + B)	Days 1-13 (variable) “Day”: 7 AM - 5:30 PM	[None]	If a Day shift is on a Friday, Saturday, Sunday, that day is off	[None]	7.0 hr
	Followed by 2 nights “Overnight”: 5 PM - 7:30 AM the following day				
	Days 16-26 (variable) “Day”: 7 AM - 5:30 PM				
	Followed by 3 nights “Overnight”: 5 PM- 7:30 AM the following day				

Note: For each group schedule, an individual will start on any day within that schedule so that there is complete coverage across the group schedule. For example, for the Q3 schedule, one person will start on “Day 1”, one on “Day 2”, and one will have the first day off because it is the end of the overnight shift. For XC-V2, one person may have only 3 Days then 3 Overnights then 10 Days then 2 Overnights

Light levels used for the simulations were 150 lux, corresponding to standard indoor room light levels, when the person was awake and 0 lux when the person was asleep.

### Model output including summary statistics

The primary metrics used were the performance and alertness outputs by the model. Summary statistics were computed for (i) each “workshift” between midnight one day and midnight the following day. These data included the scheduled work time plus 45 min before and 45 min after work time to include commuting time to and from work; (ii) work during 6 am-10 pm (“workshift-day”); and (iii) work during 10 pm-6 am (“workshift-night”). Each of these was computed for individuals and for a team schedule (e.g., the 3 people who combined cover a Q3 schedule). We then calculated the values corresponding to the 5^th^ to 95^th^ percentiles of performance or alertness across each group schedule during the entire workshift, workshift-day and workshift-night hours. We also calculated the percent of time values in the simulated schedules were less than the level of predicted performance or alertness in a habitually entrained individual after 16 h of wake and 24 h of wake. We chose these wake durations because several publications have reported that performance after those durations of wake are equivalent to being legally drunk [11, 39]. We calculated the average within each one hour bin performance and alertness for everyone working during those hours.

## Results

**Fig. 1:**
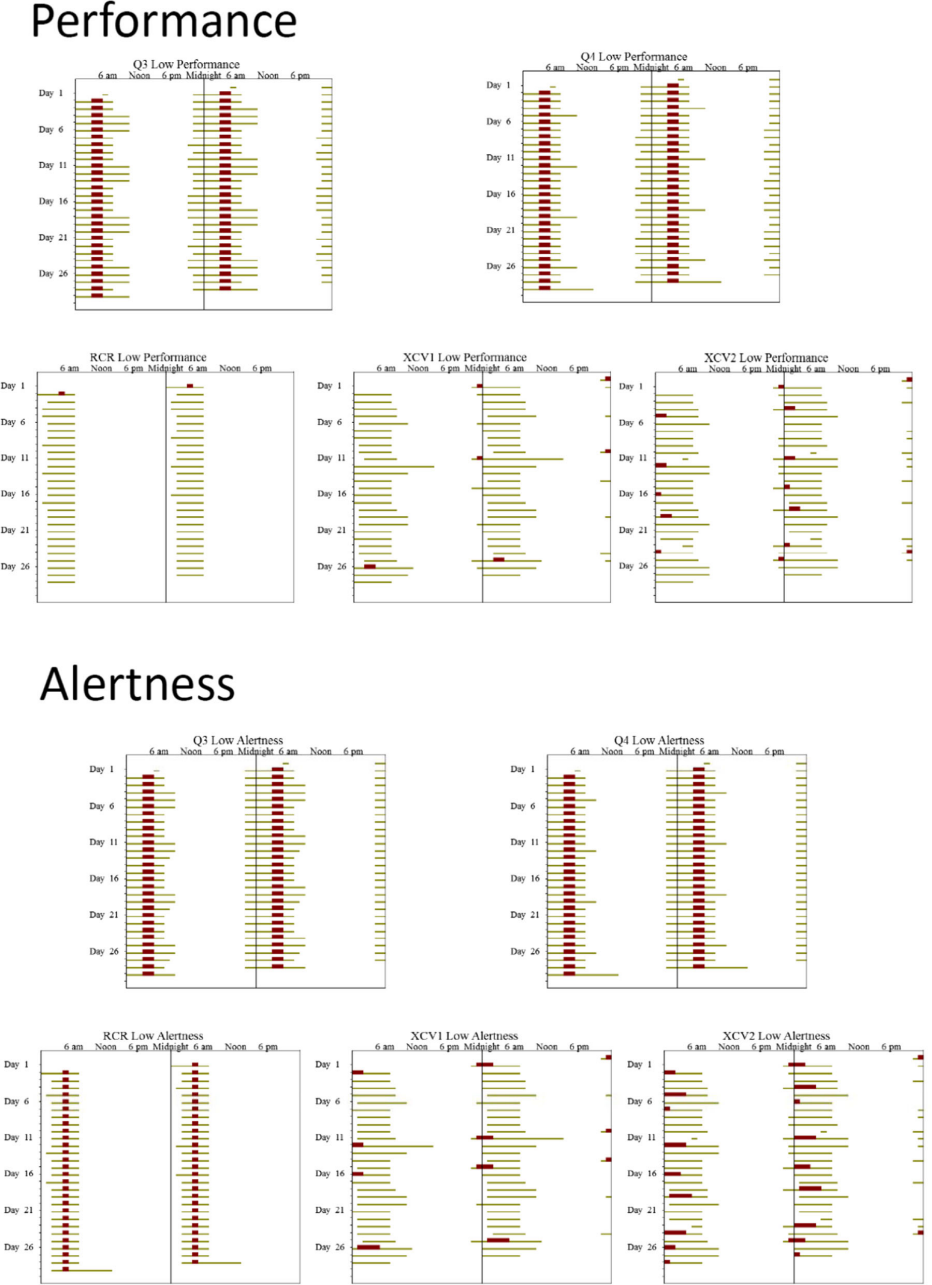
Double raster plots of the times of predicted low performance and alertness for the five schedules. In a raster plot, time in hours is plotted across the horizontal axis and time in days are plots vertically from the top. For this plot; 48 h are plotted horizontally; therefore the data on the right half of each line is duplicated on the left half of the next line. Times when performance (*top panel*) or alertness (*bottom panel*) are predicted to be less than that after 16 h (*yellow bars*) or 24 h (*red bars*) wake at habitual time are shown

Model simulation values for 16 and 24 h after awakening at habitual waketime are 81 and 51 for performance and 70 and 25 units for alertness, respectively. The hours when the average performance and alertness are less than these values are shown for each schedule in Fig. 1; yellow is for times less than predicted of 16 h awake and red for times less than predicted after 24 h of wake. Some of the daily variability is due to the reduced staffing during weekends. The model predicts that individuals are still capable of performing and feeling alert (levels corresponding to that predicted for less than 16 h of wake) at some times during most portions of the schedule. For all schedules, there is more variability across days in the amount of time spent at lower levels of performance and alertness (e.g., 50 or lower) than for predicted higher levels (data not shown).

**Fig. 2:**
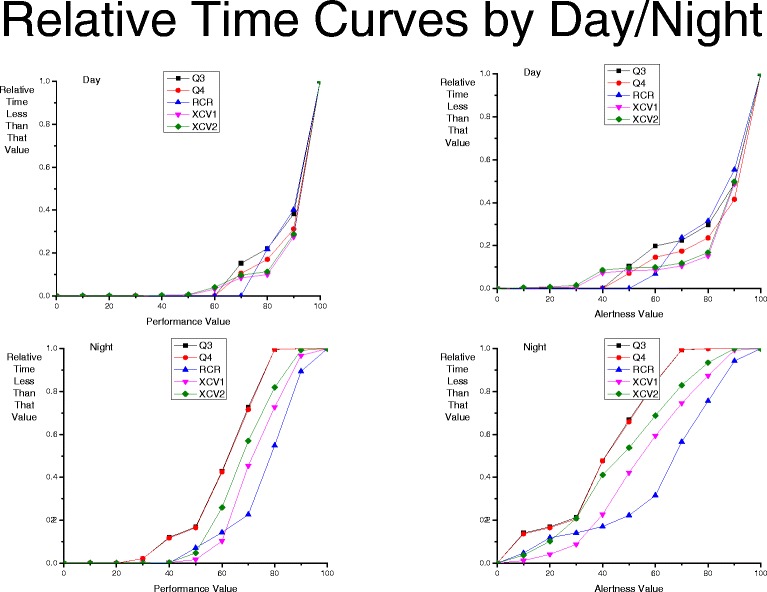
Relative Time Curves of the percent of time spent less than a performance (*left panels*) or alertness (*right panels*) value for each of the five group schedules for day work (*top panels*) and night work (*bottom panels*) hours. Note that for the night-time schedules, the Q3 and Q4 values overlap and values for only one of those schedules is easily visible

A comparison of time spent at each level of performance and alertness across the group schedules is shown in Fig. 2. More time is spent at higher levels of performance and alertness in the three intervention schedules, as is indicated by a line with more shallow slope for x-axis values less than 75, which is the approximate value at the end of 16 h of wake (e.g. right-most curves).

During the day work hours, there is almost no difference among group schedules. However, for all schedules, the performance and alertness is worse during the night - when both the circadian system and length of time awake factors both predict lower performance and alertness and when there is less attending and senior resident oversight - than during the day. The major difference in predicted performance and alertness between the schedules is during night work; all group schedules spend more time at lower values, but the three intervention schedules have much less time spent at these relatively low performance and alertness values: 10 % of time was spent at performance values less than 40 for the Q3 and Q4 schedules but ~0 % of the time was at performance values less than 40 for the three intervention schedules of RCR, XC1 and XC2. During night work hours, more time is spent with lower values (i.e., 75 or less) in predicted alertness than with lower predicted performance.

## Discussion

We found that three proposed intervention schedules that limited interns to no more than 16 consecutive hours were each predicted by our mathematical modeling tools to yield better performance and alertness than traditional Q3 or Q4 work schedules, especially during night work hours when there is less supervision. Safety, patient care, and learning of medical skills therefore would also be expected to be affected by these schedules. As residency program directors struggle to meet the changing work hour requirements for their trainees, tools such as that we have designed here can assist them in designing novel schedules that are likely to yield safer care, though as discussed below, further development of these models would help to ensure the precision and usefulness of predictions.

Per the Institute of Medicine’s 2009 recommendations, no resident should work for longer than 16 h without sleep. While the ACGME’s implementation of a 16-h limit for interns alone falls short of this recommendation, it nevertheless represents a substantive change in the structure of medical care delivery and training in the United States. In conjunction with work hour limits, the IOM’s recommendations address essential issues of supervision, workload, and handoffs that are essential to maintaining a high level of patient care. We have previously proposed that a successful transition to evidence-based work hour limits requires: (1) development of evidence based work-hour limits for physicians; (2) dissemination of best practices in safe scheduling that adhere with these limits and incorporate principles of sleep and circadian practices; and (3) development of infrastructure changes that support the implementation of shorter work hours, and promotion of a team culture [35, 36]. Examples of successful efforts to reduce resident physician work hours while implementing needed infrastructural changes are beginning to emerge [40]. Technological tools, both to help with transitions of care [41] and to aid in schedule design may help facilitate these needed changes.

The research presented in this paper is designed primarily to assist in identifying and implementing safe scheduling practices that adhere to the 2009 IOM recommendations, the 2011 ACGME limits, and principles of sleep and circadian science. Evaluating proposed schedules against the IOM’s recommendations is an essential step in our approach to ensure that collective experience of the safety community and practicing physicians accumulated by the IOM are adhered to. For example, the IOM guidelines were selected in part to reduce the exposure and implications of chronic sleep restriction (i.e., multiple days with insufficient sleep) on physician trainees [12]. The consequence of the pre-screening potential schedules for their predicted effects on performance and alertness is to reduce the number of schedule alternatives that need to be considered. The mathematical models provide a way to calculate the predicted performance by taking into account the input of the circadian and homeostatic system that includes this interaction. These nonlinear models have been developed from experimental data collected from hundreds of individuals from experiments designed to understand the relative contribution of the circadian and homeostatic systems in the prediction of performance. Thus, the mathematical models provide an efficient way for which to determine the effect of schedules on predicted circadian phase and therefore performance and alertness. The combined pre-screening of the schedules and the application of the mathematical models is designed to provide quantitative evaluation of the schedules while compensating for the limitations of mathematical models (as discussed in the Limitations section below) that in practice do not take into account all of the features of the operational environment.

An important outcome of the paper is an objective approach to evaluating schedules through the application of novel technology. Although simulation approaches cannot replace experimental approaches, the simulation results presented in this paper could provide a viable approach for determining, collecting and disseminating best scheduling practices. The efficacy of a simulation approach to evaluating resident schedules is supported by our results that demonstrate schedules consistent with the new guidelines are superior to traditional schedules and the quantitative information provides a way to compare schedule alternatives.

Our results suggest that implementation of any of the three novel schedules tested would improve performance over the Q3, or Q4 schedule. The results demonstrate that effective planning can ameliorate the exposure to circadian misalignment and extended work shifts. It also appears that, in practice, elimination of periods of poor performance and presumably periods of increased risk cannot be eliminated, but can be minimized. Field studies substantiate the notion that interventions to reduce resident work hours can improve resident alertness and patient safety. We found previously in a randomized controlled trial that implementation of a 16 h rapid cycle rotation schedule resulted in improved sleep and alertness, and decreased medical errors [3, 6]. Further high-quality studies comparing alternative scheduling interventions are needed, but preliminary evidence evaluating a range of scheduling interventions suggests that most interventions reducing or eliminating shifts over 16 h convey safety and quality of life benefits [5]. Of note, the novel schedules proposed here need not be restricted to interns; quite the contrary, the simulations would predict that such schedules would improve the performance of second-year and higher residents as well, consistent with the IOM’s 2009 recommendations.

Creating a dialog with the Boston Combined Residency Program (BCRP) in Pediatrics that included actual schedules under consideration provided realistic constraints for our residency scheduling research. Our techniques facilitate schedule simulations, although the rationale for generating different scheduling alternatives is not incorporated in our methods. Simulation results provide an objective way to assess the biological implications of schedules under consideration. Initially, our dialog with residency program officials involved defining schedule constraints and assessing scheduling alternatives. Following a meeting in which we shared our results, the BCRP selected schedules that were assessed to be best by our methods. Such a collaboration served not only to promote dissemination of best predicted schedules, but an ongoing relationship in which education in sleep and circadian biology will be provided to residents, and in which the door is open for future refinements of work schedules.

Mathematical models have been used to predict performance and alertness in experimental settings. However, comparison with actual real-work risk is rare. The results of applying the SAFTE model to train operator schedules and then relating those to the risk and cost of train accidents revealed a linear relationship with higher risk and cost associated with worse predicted performance [18]. Application of the SAFTE model to study the predicted performance of orthopedic resident physicians demonstrated that they often work in an impaired condition, but this study did not quantify the potential to address this risk through implementation of alternative schedules [42]. A different model applied to car accident risk also found a predicted relationship [19]. Our model was able to predict use of sleep medications in space [27], an indication of sleeping problems that may produce waketime performance and alertness deficits. A major difficulty however, is in applying the model to individuals, since the individual risk of most severe outcomes is sufficiently low that large numbers of people typically need to be studied to detect significant effects.

### Limitations

Our study and modeling tools have several limitations. First, it is important to note that the predictions made to date using the tools developed here are sensitive to the amounts and timing of sleep obtained, so the findings comparing our intervention schedules to traditional schedules may not be generalizable to all instances of these general schedule types. Mathematical modeling is, however, capable of accounting for these nuances and evaluating the predicted effects of even subtle changes to sleep and scheduling parameters, within the constraints of the scheduling software. Secondly, the current CPSS software does not include a term that accounts for chronic sleep restriction (multiple nights with insufficient sleep), which could potentially modify both the precise results predicted here and the assessment of the relative merits of one schedule vs. another. While we would not anticipate that incorporation of a chronic sleep restriction term would change our principal finding that predicted performance on the three intervention schedules was superior to performance on traditional Q3 and Q4 schedules (given the large magnitude of differences seen between intervention and traditional schedules), it could affect subtler differences in predicted performance, such as the relative merits of the three intervention schedules, each of which would be anticipated to be differentially affected by chronic sleep restriction. Consequently, we believe it important not to over-interpret the results presented here, but rather to view them in the context both of the emerging state of modeling science, and the broader literature on resident sleep, performance, and safety. Lastly, the application of mathematical models to evaluate schedules is only one approach to mitigate risk. The fatigue and safety community stress the importance of a fatigue management program that includes buy in from stake holders, training, a fatigue management plan, and may include technological components integrated within the environment [43, 44]. We assumed that predicted performance decrements may correspond to an increase in occupational errors. There are studies supporting this assumption (cited above); however, prediction of individual risk at a specific time is not yet accurate.

## Conclusion

Mathematical modeling is a useful tool for evaluating residency schedules. The residency program we studied implemented a schedule we assessed and now educates residents on sleep deprivation, sleep hygiene, and the importance of napping before night shifts. Using the mathematical model, we can provide quantitative evidence to be used in schedule reform.

Nationally, there are approximately 100,000 interns and residents, distributed throughout thousands of residency programs in tertiary care and community hospitals nationwide. Resident physicians care for a substantial proportion of the 36 million patients seen at U.S. hospitals each year. Improved resident physician performance could therefore result in improved quality of care for millions of patients annually. In addition to the direct effect on residents and their patients, the methods developed in this project have the potential to provide objective assessment of current work schedule policies and to inform work hour policies in other occupations. A novel application of the tool could be a standard format for monitoring and evaluating residency work schedules. Schedules designed with a standard tool could form the basis for a national archive of approved schedules and provide an initial screening of residency program compliance with appropriate software tools. Such an approach might be pursued collaboratively with the ACGME, funding from the Agency for Healthcare Research and Quality (AHRQ), Residency Programs, and private scheduling software companies. Lastly, the ability to explicitly schedule specific types of work blocks (i.e. rotation, clinic and call) could provide a framework designed to assess different schedule components either though task specific models or comparison to data collected in the field. Using data collected during training rotations to generate operationally appropriate models could provide simulation estimates tailored to the residency environment.

## Abbreviations

ACGME: U.S. Accreditation Council for Graduate Medical Education
AHRQ: Agency for Healthcare Research and Quality
BCRP: Boston Combined Residency Program in Pediatrics
CPSS: Circadian Performance Simulation Software
HWHHSG: Harvard Work Hours, Health, and Safety Group
NASA: National aeronautics and space administration
PGY1: 1^st^ year interns
PGY2: 2^nd^ year residents
Q3: Three-day rotation
Q4: Four-day rotation
RCR: Rapid cycle rotation
XC: Cross-cover rotation
XC-V1: Cross-cover rotation version 1
XC-V2: Cross-cover rotation version 2
